# Prediction of mid-term outcome after cryo-balloon ablation of atrial fibrillation using post-procedure high-sensitivity troponin level

**DOI:** 10.5830/CVJA-2015-027

**Published:** 2015

**Authors:** Tolga Aksu, Tümer Erdem Guler, İsmail Erden, Sukriye Ebru Golcuk, Kıvanç Yalin

**Affiliations:** Department of Cardiology, Derince Education and Research Hospital, Kocaeli, Turkey; Department of Cardiology, Derince Education and Research Hospital, Kocaeli, Turkey; Department of Cardiology, Derince Education and Research Hospital, Kocaeli, Turkey; Department of Cardiology, Faculty of Medicine, Istanbul University, Istanbul, Turkey; Department of Cardiology, Faculty of Medicine, Istanbul University, Istanbul, Turkey

**Keywords:** ablation, atrial fibrillation, cryo-balloon, troponin, recurrence

## Abstract

**Objective:**

High-sensitivity troponin I (hsTnI) assays lead to, among other things, improvement in the detection of myocardial injury and improved risk stratification of patients with atrial fibrillation (AF). The aim of this study was to investigate the association between post-procedure cardiac biomarkers and clinical outcome in patients undergoing cryo-balloon ablation (CA) for AF.

**Methods:**

A total of 57 patients (mean age 55.1 ± 12.2 years, 50.9% female) with symptomatic paroxysmal AF underwent the CA procedure. Two hundred and twenty-eight pulmonary veins (PVs) were attempted for pulmonary vein isolation (PVI) with a second-generation cryo-balloon. hsTnI, CK-MB mass and myoglobin samples were prospectively obtained before and 24 hours after ablation.

**Result:**

At a mean follow up of 214.6 ± 24.3 days, the probability of being arrhythmia free after a single procedure was 86%. Post-ablation hsTnI (*p* = 0.001), left atrial (LA) diameter (*p* = 0.002), duration of AF (*p* = 0.002), mean minimal temperature of the left superior pulmonary vein (*p* = 0.005), and age (*p* = 0.021) were associated with increased AF recurrence rate. On multivariate analysis, lower hsTnI level was the only independent predictor for AF recurrence (*p* = 0.012). Post-ablation hsTnI levels lower than 4.40 ng/ml predicted AF recurrence during follow up, with a sensitivity of 86% and a specificity of 96%.

**Conclusion:**

It is well recognised that the PV antrum contributes to initiation and/or perpetuation of AF. A lower postablation hsTnI level may predict an increased AF recurrence rate, suggesting inadequate ablation of the PV antrum. This may be used as a non-invasive marker to predict the outcome of AF.

## Objective

Atrial fibrillation (AF) is the most common cardiac arrhythmia, with an estimated prevalence of 1–3%.^1^,^2^ The development of AF requires both a trigger and a susceptible substrate. The most common trigger for AF is the myocardial sleeve of the left atrium (LA), which extends into the pulmonary veins (PVs).^3^ However, the PV antrum also contributes to initiation and/or perpetuation of AF.^4^,^5^ Therefore, ablation of these sites, particularly pulmonary vein isolation (PVI), remains the cornerstone of AF ablation procedures.^6^

However, in 25–50% of patients, PVI may not be sufficient due to greater extension of atrial fibrosis, or PV reconnection as a result of non-transmural lesion formation.^5^-8 For this reason, in addition to PVI, the creation of different ablation lines (roof, posterior line and mitral isthmus) in the left atrium (LA) has been proposed for successful radiofrequency (RF) catheter ablation.9-12

Application of RF energy leads to the release of myocardial injury markers immediately after the ablation procedure and the level of released cardiac biomarkers are linked to the extent of ablation-induced cardiac lesions.^13^ To determine the size of effective ablation lesions comprising different energy sources, many authors have used a variety of cardiac biomarkers.^14^-^25^ Contrasting data exist about myocardial injury biomarker trends after cryo-balloon ablation (CA) procedures.^15^-^19^

In our previously published abstract, we demonstrated that cryo-balloon ablation may be linked to significant decrease in left atrial potentials adjacent to the PVs, particularly on the posterior wall of the LA, compared to the RF-based PVI procedure in patients with long-standing persistent AF, which limited the sites of ablation.^26^ To date, it is unknown whether myocardial injury biomarkers could predict the extent of lesion formation at long-term follow up.

We aimed to investigate the sensitivity and specificity of postprocedural cardiac biomarker levels for predicting recurrence of AF in patients undergoing CA for paroxysmal AF, and to discuss the pathophysiological basis of these relationships – inadequate left atrial ablation or unsuccessful PVI. Other potential predictors of AF recurrence were also evaluated in the same population.

Isolation of all PVs was the procedural endpoint. The primary endpoints of the study were (1) comparison of peak biomarker release in patients with/or without AF recurrence before and after the procedure, and (2) comparison of procedural parameters in patients with/or without AF recurrence. The secondary endpoint was other potential predictors of recurrent atrial tachycardia of more than 30 seconds during mid-term follow up of six months without a blanking period.

## Methods

The study population consisted of 57 consecutive patients who underwent PVI with the cryo-balloon technique for 12-lead verified, symptomatic and drug-refractory paroxysmal AF. Patients whose episodes of AF had self-terminated within seven days were defined as paroxysmal AF. The indication for ablation was based on the guidelines.6 Detailed inclusion and exclusion criteria for patients are outlined in Table 1.

**Table 1:** Study inclusion and exclusion criteria

*Inclusion criteria*
Patients age ≥ 18 years
Paroxysmal AF (AF that terminates spontaneously or with intervention within 7 days of onset)
Symptomatic and drug refractory (at least one anti-arrhythmic) AF
At least three episodes of AF must have been documented by ECG or Holter before the procedure
Patients must be on continuous anticoagulation with warfarin (INR 2–3) for > 4 weeks prior to the ablation
Patients must be able and willing to provide written informed consent to the procedure
*Exclusion criteria*
Previous abdominal surgical procedures
History of either acute or chronic neuropathies
Usage of drugs that affect gastrointestinal motility
Persistent or permanent AF
Inadequate anticoagulation as defined in the inclusion criteria
Left atrial thrombus on transoesophageal echo prior to the procedure
Contra-indications to any anticoagulant
Previous AF ablation procedure
Left atrial size > 55 mm
Left ventricular ejection fraction < 30%
Congestive heart failure with New York Heart Association class IV

Symptomatic severity of the patients was recorded according to the European Heart Rhythm Association (EHRA) score. CHA_2_DS_2_-VASc scores were calculated for each patient based on the relevant guidelines.^27^ Written informed consent was obtained from all patients before the procedure. The local ethics committee approved the study.

Pre-procedural evaluation: standard transthoracic echocardiography (TTE) was performed in all patients to evaluate left atrium diameters and to rule out structural abnormality. In all patients, left atrial thrombus was ruled out by transoesophageal echocardiography (TEE) prior to the procedure. All patients were anticoagulated with warfarin to maintain an international normalised ratio (INR) of 2–3 for at least four weeks prior to the procedure. Warfarin was interrupted before the procedure. The procedures were done if the INR value was < 1.5. Antiarrhythmic drugs were discontinued five half-lives before the procedure.

Blood sampling and biomarker measurements: blood samples were obtained during venous puncture before the procedure and a further one and 24 hours after ablation. The blood level of hsTnI was measured in frozen EDTA plasma samples using the current version of the AccuTnI assay (Beckman Coulter Inc, Fullerton, CA, USA). Serum CK activity was determined using an analyser Integra (Roche). The reference ranges in our laboratory for the cardiac markers were as follows: CK-MB mass, 0.5–5 ng/ml; myoglobin, 0–113 ng/ml; and hsTnI, 0.0–0.06 ng/ml. Cardiac hsTnI cut-off values for the diagnosis of myocardial infarction (0.06 ng/ml) were accepted as pathologically increased.

Ablation procedure: the procedure was performed under local anesthesia. Trans-septal punctures were performed under fluoroscopic guidance only. After trans-septal puncture, intravenous heparin was used to maintain an activated clotting time of 300 to 400 seconds. A single or double trans-septal puncture was performed using a conventional circumferential mapping catheter (Inquiry^TM^, Optima^TM^, St Jude Medical, Sylmar, CA, USA) or the customised mapping catheter (Achieve^TM^, Medtronic, Minneapolis, MN, USA). Positioning of the 28-mm cryo-balloon catheter (Arctic Front Advance^TM^, Medtronic, Minneapolis, MN, USA) was achieved using a guidewire and a 12-Fr steerable sheath (Flexcath Medtronic Minneapolis, MN, USA). While delivering cryo-energy to the right PVs, a 6-F decapolar coronary sinus (CS) catheter or a quadripolar diagnostic catheter was positioned in the superior vena cava for phrenic nerve stimulation.

Before each freeze, the grade of occlusion (semi-quantitative scale from 1 = poor occlusion to 4 = perfect occlusion) was quantified with an injection of contrast medium.^28^ After confirmation of PV occlusion by contrast injection, the 240-second freezing cycle was initiated. After two freezing cycles, PVI was assessed using a circumferential mapping catheter. If PVI was not achieved within five attempts, the customised mapping catheter was exchanged for a stiffer wire (Amplatz Ultra Stiff, COOK Medical Inc). Isolation of PVs was defined as the presence of both entrance and exit block.

In all patients, rapid atrial pacing from the distal tip of the CS catheter was used to induce AF after the procedure. If AF could not be induced or sustained for longer than one minute by rapid atrial pacing, an infusion of isoproterenol (10 mcg/min) was used to sustain AF. Complex fractionated electrogram (CFE) mapping was performed to detect any focal source except for PVs, if the AF persisted for more than one minute. CFE mapping using an automated algorithm (Ensite NavX, St Jude Medical) was performed in the LA, CS and right atrium. The technique for CFE mapping using automated mapping software has been described and validated previously.^29^ Patients in whom CFE was detected outside the LA were excluded from the study.

For RF ablation of CFE, an open irrigated-tip catheter with a 3.5-mm tip electrode (ThermoCool, Biosense Webster) was used in conjunction with a three-dimensional electro-anatomical mapping system (NavX Fusion, St Jude Medical). The energy of the RF was delivered with power of up to 35 W and a maximum temperature of 43°C. The endpoint for CFE ablation was (1) elimination of all CFE sites in the LA or termination of AF, and (2) non-inducibility of AF post ablation with the same protocol.

Post-procedural evaluation: TTE was performed immediately after the procedure to exclude the presence of pericardial effusion. All patients were followed up for at least 12 hours in the intensive care unit. Patients were then discharged provided that their clinical status was stable. Oral anticoagulation was initiated on the evening of ablation unless pericardial effusion was detected, and continued for at least three months after the procedure. The patients presenting with arrhythmia-related symptoms in the first three months were treated with anti-arrhythmics (amiodarone in three cases and propofenone in one case).

Regular follow up consisted of out-patient clinic visits one, three and six months after the procedure. It included a detailed history for arrhythmia-related symptoms (palpitations, chest discomfort, fatigue and dizziness), a physical examination, 12-lead ECG and 24-hour Holter monitoring.

The need for further oral anticoagulation was evaluated in the third month based on the CHA_2_DS_2_-VASc score. Outcome was measured as per the guidelines in the recent consensus document.^6^ Any episode of AF, atrial flutter, or atrial tachycardia lasting for at least 30 seconds was defined as recurrence.^6^ A blanking period was not considered for the study. Any recurrence in the first three months was classified as early recurrence, whereas recurrence after this period was considered late recurrence. Only patients with at least six-month follow ups were included in the study.

Patients with late AF recurrence underwent redo-procedures to evaluate the cause of recurrence, following similar preparatory steps to those during the index procedure. A diagnostic catheter was used to evaluate the status of isolation or reconnection in each vein.

None of the patients had clinical signs of coronary ischaemic episodes either prior to or at the end of the procedure. There were no changes in the ST-segment when comparing ECG tracings before, during and after the procedure.

## Statistical analysis

SPSS 17.0 software (IBM Corp, Armonk, NY, USA) was used for statistical analysis. All quantitative variables with a normal distribution were reported as mean ± standard deviation, and compared using the Student’s *t*-test. For values with non-normal distribution, comparison was performed with the Mann–Whitney *U*-test. For the descriptive variables comparison, the Pearson χ^2^ or Fisher’s exact tests were used, when appropriate.

The independent association of clinical variables with recurrence was assessed using multivariable linear regression. A Kaplan–Meier analysis was used to analyse the recurrent atrial tachycardia‐free survival after cryo-ablation. A *p*-value < 0.05 was considered statistically significant. Receiver-operator characteristic (ROC) analysis was performed on significant predictors to calculate the accuracy and other diagnostic parameters, and to determine a cut-off point at the maximum sum of sensitivity and specificity.

## Results

**Figure 1.**
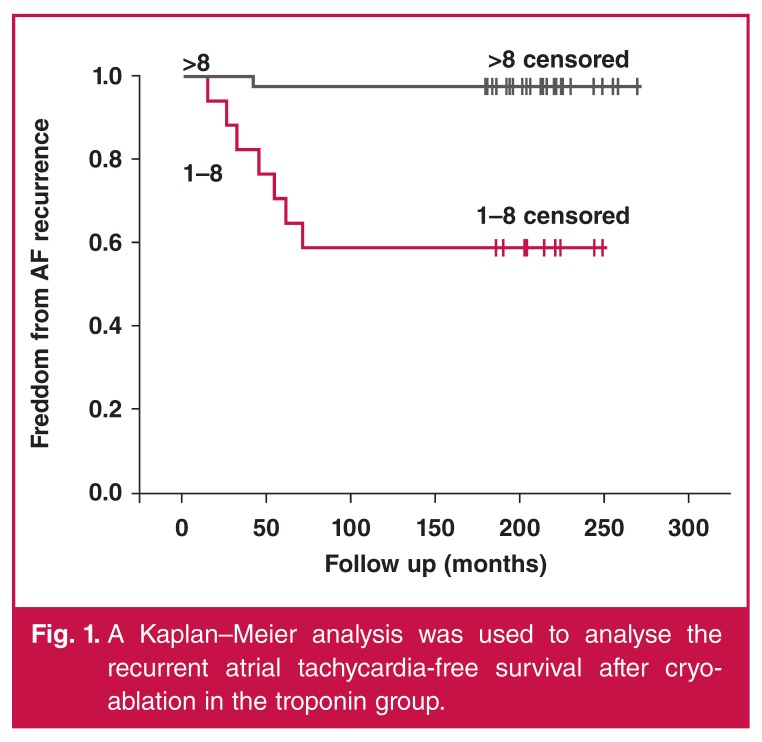
A Kaplan–Meier analysis was used to analyse the recurrent atrial tachycardia‐free survival after cryoablation in the troponin group.

Baseline characteristics and demographic features of the patients are presented in Table 2. After a mean follow-up period of 214 ± 24 days (range 180–274), early recurrence was observed in five (8.77%) patients and late recurrence in two (3.50%) (Fig. 1). Patients with AF recurrence (group 1) were significantly older (65 ± 15 vs 54 ± 1 years, *p* = 0.002), had larger left atrial diameters (46.2 ± 4.3 vs 40.7 ± 4.1 mm, *p* = 0.002) and longer duration of AF (6.7 ± 4.5 vs 3.5 ± 1.9 years, *p* = 0.002) than patients with no recurrence (group 2). The other baseline demographics were comparable between the groups.

**Table 2:** Baseline characteristics and demographic features of the study population (*n* = 57)

	*Total (n = 57)*	*Recurrence (–) (n = 50)*	*Recurrence (+) (n = 7)*	*p-value*
Failed anti-arrhythmics (*n*)				
Amiodarone	13	11	2	0.630
Propofenone	27	23	4	0.594
βb or CKB	17	15	2	0.409
Age, years (mean ± SD)	55.1 ± 12.13	54 ± 1	65 ± 15	0.021
Gender, female, *n* (%)	29 (50)	26 (52)	3 (42)	0.658
BMI, kg/m^2^	24.8 ± 3.7	24.8 ± 3.6	24.7 ± 3.7	0.126
Diabetes mellitus, *n* (%)	10 (17)	9 (18)	1 (14)	0.195
Hypertension, *n* (%)	25 (43)	21 (42)	4 (57)	0.451
CAD, *n* (%)	9 (15)	8 (16)	1 (14)	0.457
Smoking, *n* (%)	28 (49)	25 (50)	3 (42)	0.702
Duration of AF history, years	3.9 ± 2.6	3.5 ± 2.5	6.7 ± 4.5	0.002
LA diameter, mm	41.32 ± 4.51	40.72 ± 4.16	46.23 ± 4.36	0.002
LVEF, %	59.23 ± 5.12	59.48 ± 4.78	56.42 ± 5.56	0.146
CHA2DS2-VASc score, mean ± SD	1.3 ± 1.17	1.3 ± 1.11	1.7 ± 1.60	0.414
EHRA score, mean ± SD	2.45 ± 0.56	2.44 ± 0.54	2.57 ± 0.78	0.573
Follow-up time, days, mean ± SD	214 ± 24	212 ± 23	213 ± 25	0.117

AF, atrial fibrillation; βb, beta-blocker; BMI, body mass index; CKB, Ca channel blocker; CAD, coronary artery disease; EHRA, European Heart Rhythm Association; LA, left atrium; LVEF, left ventricular ejection fraction; SD, standard deviation, *p* < 0.05.

All patients were in sinus rhythm at the beginning of the procedure. The procedural endpoint of PVI was reached in all patients. A left-sided common ostium was seen in two patients. Four patients were in AF at the end of cryo-ablation; the regions of CFE were located within the PV antrum or posterior LA wall in these four patients, and additionally on the LA roof in three patients, and high on the LA septum in one patient. Sinus rhythm was achieved in all four patients after CFE ablation. At the end of CFE ablation, repeat induction was attempted in all four patients but neither AF nor atrial tachyarrhythmia was induced after CFE ablation in any of them.

Mean procedure time and mean duration of energy delivery were comparable in the groups, but mean minimal temperature was higher in group 1 than in group 2 (–46.21 ± 1.55 vs –49.14 ± 3.04°C, *p* = 0.016). This difference was caused by higher mean minimal temperature in the left superior pulmonary vein (Table 3). Mean pre-procedural ACT levels and mean ACT level during the procedure were comparable between the groups.

**Table 3:** Procedure-related data (*n* = 57)

	*Total (n = 57)*	*Recurrence (–) (n = 50)*	*Recurrence (+) (n = 7)*	*p-value*
Minimal temperature (°C)				
LSPV	50.14 ± 3.51	–50.63 ± 3.46	–46.71 ± 1.11	0.005
LIPV	48.12 ± 3.94	–48.46 ± 4.04	–45.71 ± 1.97	0.084
RSPV	51.45 ± 3.67	–51.80 ± 3.74	–49.45 ± 1.91	0.053
RIPV	45.45 ± 3.59	–45.74 ± 3.59	–43.42 ± 3.10	0.111
Occlusion grade				
LSPV	3.82 ± 0.38	3.88 ± 0.32	3.42 ± 0.53	0.764
LIPV	3.77 ± 0.42	3.84 ± 0.37	3.28 ± 0.48	0.605
RSPV	3.98 ± 0.13	3.98 ± 0.14	3.99 ± 0.03	0.408
RIPV	3.80 ± 0.39	3.82 ± 0.38	3.71 ± 0.48	0.143
Freezing duration (min)				
LSPV	8.42 ± 1.40	8.40 ± 1.45	8.57 ± 0.97	0.766
LIPV	8.75 ± 1.76	8.80 ± 1.84	8.42 ± 1.13	0.602
RSPV	8.24 ± 0.82	8.28 ± 0.88	8.80 ± 0.96	0.408
RIPV	10.15 ± 3.31	9.92 ± 3.20	11.85 ± 3.80	0.141
Number of applications				
LSPV	2.15 ± 0.49	2.14 ± 0.49	2.28 ± 0.48	0.462
LIPV	2.28 ± 0.61	2.30 ± 0.64	2.14 ± 0.37	0.537
RSPV	2.08 ± 0.28	2.09 ± 0.30	2.13 ± 0.55	0.396
RIPV	2.80 ± 1.23	2.74 ± 1.22	3.28 ± 1.25	0.274

LIPV, left inferior pulmonary vein; LSPV, left superior pulmonary vein; RIPV, right inferior pulmonary vein; RSPV, right superior pulmonary vein. *p* < 0.05.

Complications related to CA procedures included a major haematoma in the groin in one patient, transient phrenic nerve paralysis in four patients, a gastroparesis in seven patients and a transient ischaemic attack, which had resolved the following day, in another patient. There was no correlation between post-procedural hsTn values and the number of trans-septal punctures or inserted femoral sheaths.

**Figure 2.**
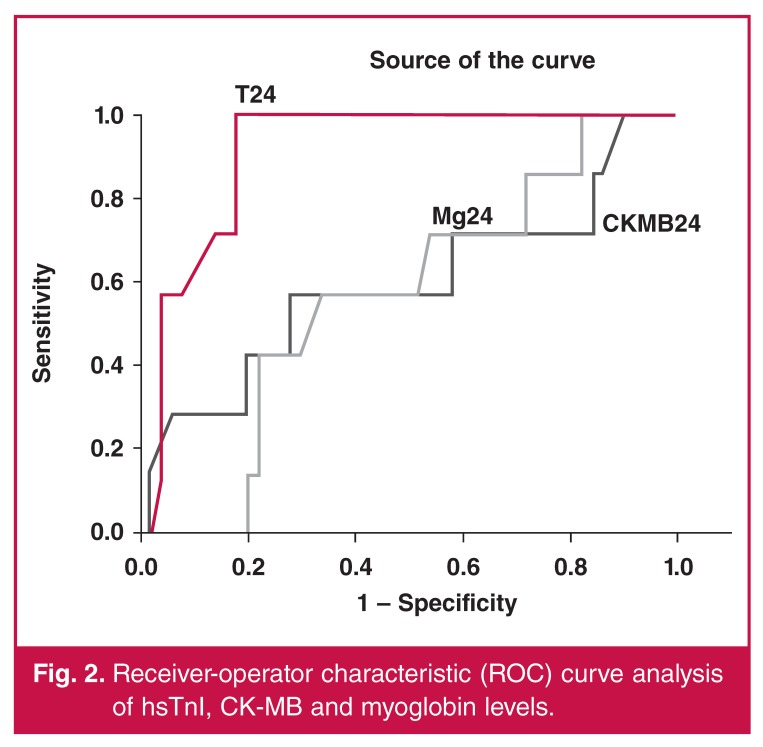
Receiver-operator characteristic (ROC) curve analysis of hsTnI, CK-MB and myoglobin levels.

**Figure 3.**
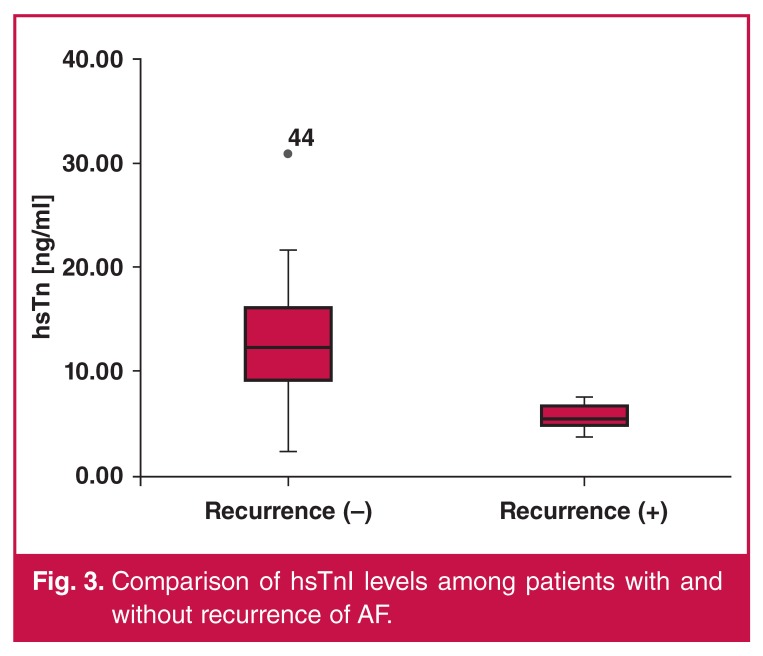
Comparison of hsTnI levels among patients with and without recurrence of AF.

In both groups, no patients showed pathological values for hsTnI, CK-MB mass, or myoglobin levels at baseline. Postprocedure blood tests were performed at about 26 ± 2.4 hours after the end of the procedure and pathological values for hsTnI were recorded in 100% of patients, with a median value of 11.75 ± 5.25 ng/ml. CK-MB mass was above the cut-off threshold in 54 of 57 patients (94.73%) patients, and myoglobin was above the cut-off threshold in 17 of 57 (29.82%) patients (Fig. 2). Mean hsTnI levels were significantly lower in group 1 (Table 4, Fig. 3). Blood levels of CK-MB and myoglobin were slightly higher in group 1 but these differences did not reach statistical significance (Table 4).

**Table 4:** Cardiac biomarker data (*n* = 57)

	*Pre-procedure (n = 57)*		*Post-procedure (n = 57)*	
	*Recurrence (–)*	*Recurrence (+)*	*Recurrence (–)*	*Recurrence (+)*
Troponin	0.01 ± 0.01	0.008 ± 0.007	12.57 ± 5.06	5.90 ± 1.42*
Creatine kinase	2.02 ± 0.97	2.43 ± 1.25	30.36 ± 21.37	36.88 ± 21.12
Myoglobin	22.27 ± 8.91	15.52 ± 3.29	72.99 ± 20.88	82.14 ± 30.31

*p < 0.001.

After multivariate analysis including duration of AF, postprocedure hsTnI level and left atrial diameter as covariates, only post-procedure hsTnI level remained a significant predictor for ablation recurrence. There was no correlation between post-CA biomarker levels and mean minimal temperature in the PVs.

Both patients with late recurrence underwent redo electrophysiological studies (EPS) to determine the possible cause of recurrence. One of these patients underwent CFE ablation due to persistence of AF after CA. The induction of AF was achieved by rapid atrial pacing, as mentioned above. Conventional EPS showed no reconnection of the PVs. Termination of AF was achieved during CFE ablation at the anterior wall of the LA in one patient and on the LA roof in the other.

## Discussion

The main findings of this study were that cryo-ablation for paroxysmal AF resulted in an increase in hsTnI levels. In addition, hsTnI level was the only independent predictor of AF recurrence in multivariate analysis.

The STOP-AF trial is the only randomised study that compared treatment efficacy of cryo-balloon ablation versus antiarrhythmic drugs for paroxysmal AF. After 12 months, nearly 70% of the patients treated with the cryo-balloon remained free from AF, compared to only 7.3% on drug therapy.^25^ The success rate of treatment was higher with CA than with standard RF ablation.^30^

Aytemir *et al.*^31^ studied the predictors of AF recurrence in patients who underwent cryo-balloon ablation for paroxysmal AF. In this study, freedom from AF after a single ablation procedure was 68.53 and 90.83% in patients undergoing PVI with first- and second-generation cryo-balloon catheters, respectively. Left atrial diameter, early AF recurrence and second-generation cryo-balloon catheter use were independent predictors for late AF recurrence.

In our study, we used second-generation cryo-balloon catheters in all cases and 88% of patients were free of AF recurrence. In patients with AF recurrence, the LA diameter was larger and the duration of AF was longer. After multivariate analysis, these two parameters were not found to be independent predictors of recurrence.

The role of troponin release after CA of PVs for paroxysmal AF in predicting ablation outcome is not clear. Cardiac biomarkers have been used to estimate myocardial lesion size after ablation procedures with different energy sources. Del Rey *et al.*^32^ demonstrated that RF ablation increased troponin levels in almost all patients, whereas other cardiac biomarkers remained within health-related reference limits. It has also been shown that increase in biomarker levels and the amount of myocardial damage after RF catheter ablation depend on the number of RF pulses and the site of ablation.^22^

Increase in myocardial injury biomarker levels after CA was first described by Oswald *et al.*^15^ In patients with atrial flutter, CA showed significantly higher troponin levels following ablation compared to RF ablation, with declining levels the following day. They observed equal findings for CK and CK-MB levels, both significantly higher in the CA group.

Comparison of troponin increases after ablation procedures for AF with RF or cryo-energy is controversial. Kühne *et al.*.^16^ compared troponin release in patients undergoing CA and RF ablation. In their study, post-procedural troponin levels were higher in the RF ablation group. The study by Siklody *et al.*^20^ revealed no significant differences in myocardial injury markers between patients treated with CA or RF ablation. In the same field, Schmidt *et al.*^20^ compared RF ablation and CA for their impact on markers for myocardial injury. They demonstrated that CA causes significantly higher troponin release compared to RF ablation.

In our study, CA resulted in a larger troponin increase compared to previous studies using RF ablation.^12^,^23^,^24^ To the best of our knowledge, our study is the first that shows the prognostic role of hsTnI levels in patients undergoing CA for paroxysmal AF. Our study revealed that lower post-procedural hsTnI level is an independent predictor of AF recurrence.

Although we also analysed other myocardial injury markers, we found only hsTnI level to be a predictor of AF recurrence. This may be related to the better sensitivity of hsTnI to show myocardial damage than any other markers of injury.

Bordignon *et al.*^33^ compared myocardial biomarker release using first- and second-generation cryo-balloons. They revealed that cumulative freezing time was related to biomarker release. In our study, there was no correlation between biomarker release and procedural data.

Single-procedure success rates of PVI by RF ablation in patients with paroxysmal AF remain unsatisfactory. Although PVI is the main target in paroxysmal AF, substrate abnormality in the PV antrum may play a critical role in the AF mechanism. Additional ablation of the PV antrum after PVI may increase the efficacy of the procedure.^26^ Higher troponin release with CA may be linked to larger ablation damage in the LA compared to the RF-based PVI procedure. This finding may explain the potential advantage of CA beyond PV isolation.

Preliminary results of our unpublished data on patients with long-standing persistent AF showed that CA of the PVs resulted in a significant decrease in the CFE area.^26^ This contributary role may be predominantly on the posterior wall of the LA due to its vicinity.

## Limitations

Our study has several limitations. A major limitation is the relatively small sample size. Another limitation is the poor relationship between biomarker measurement and lesion region, and we did not find a casual relationship. The mode of follow up, which was performed only by 24-hour Holter monitoring or occasional event-driven ECG is a further limitation, and clinical judgment may be questionable. The increase in hsTn levels after ablation was not region specific and it may not indicate ablation of the critical site maintaining the arrhythmia.

Although two patients underwent redo EPS to define the exact electrophysiological cause of recurrence, EPS evaluation was unfortunately not performed on all patients with recurrence. Therefore we could not conclude whether all recurrence was associated with inadequate substrate ablation in patients with lower post-CA hsTn levels.

Although patients in whom AF persisted at the end of CA underwent CFE mapping, the localisation of CFE may not predict the exact focus of triggers in patients with paroxysmal AF. Given the difficulty in precisely locating and ablating these triggers, an alternative approach that simply seeks to electrically isolate the PV from the LA seems logical.

Evaluation of histopathological data would be the gold standard to assess the extent and localisation of ablation lesions. However, the requirement for animal or *in vitro* studies constitutes the pivotal problem in this evaluation. Cardiac magnetic resonance imaging after using a cryo-balloon in patients with/or without recurrence may contribute to providing distinct information with regard to cryo-thermal cardiac lesions and associations between biomarker release, and should be a field for future research.

## Conclusion

Despite these limitations, the results of this study indicate that lower post-procedural hsTn level was associated with higher recurrence rates and may be linked to inadequate atrial ablation by cryo-balloon catheter.
